# The interferon gamma gene polymorphism +874 A/T is associated with severe acute respiratory syndrome

**DOI:** 10.1186/1471-2334-6-82

**Published:** 2006-05-04

**Authors:** Wai Po Chong, WK Eddie Ip, Gloria Hoi Wan Tso, Man Wai Ng, Wilfred Hing Sang Wong, Helen Ka Wai Law, Raymond WH Yung, Eudora Y Chow, KL Au, Eric YT Chan, Wilina Lim, JS Malik Peiris, Yu Lung Lau

**Affiliations:** 1 Department of Paediatrics and Adolescent Medicine, The Hong Kong Jockey Club Research Centre, The University of Hong Kong, Pokfulam, Hong Kong SAR, China; 2 Department of Pathology, Pamela Nethersole Youde Hospital, Hong Kong SAR, China; 3 Department of Pathology, United Christian Hospital, Hong Kong SAR, China; 4 Princess Margaret Hospital, Hong Kong SAR, China; 5 Queen Mary Hospital, The University of Hong Kong, Hong Kong SAR, China; 6 Government Virus Unit, Department of Health, Hong Kong SAR, China; 7 Department of Microbiology, The Hong Kong Jockey Club Research Centre, Pokfulam, The University of Hong Kong, Hong Kong SAR, China

## Abstract

**Background:**

Cytokines play important roles in antiviral action. We examined whether polymorphisms of *IFN-γ*,*TNF-α *and *IL-10 *affect the susceptibility to and outcome of severe acute respiratory syndrome (SARS).

**Methods:**

A case-control study was carried out in 476 Chinese SARS patients and 449 healthy controls. We tested the polymorphisms of *IFN-γ*,*TNF-α *and *IL-10 *for their associations with SARS.

**Results:**

*IFN-γ *+874A allele was associated with susceptibility to SARS in a dose-dependent manner (*P *< 0.001). Individuals with *IFN-γ *+874 AA and AT genotype had a 5.19-fold (95% Confidence Interval [CI], 2.78-9.68) and 2.57-fold (95% CI, 1.35-4.88) increased risk of developing SARS respectively. The polymorphisms of *IL-10 *and *TNF-α *were not associated with SARS susceptibility.

**Conclusion:**

*IFN-γ *+874A allele was shown to be a risk factor in SARS susceptibility.

## Background

Severe acute respiratory syndrome (SARS) is an infectious disease caused by SARS coronavirus [1] with >8000 cases and 774 deaths reported in 2003 [2]. Much progress has been made in understanding SARS coronavirus but the pathogenesis is still unclear [3]. It was reported that old age, diabetes mellitus and heart disease were risk factors for adverse prognosis of SARS [4-6], however, little is known about the contribution of genetic factors. We have demonstrated that genetic haplotypes associated with low serum mannose-binding lectin (MBL) were associated with SARS [7] and our findings were recently replicated [8]. Recently, homozygotes for *CLEC4M *tandem repeats were reported to be less susceptible to SARS in Hong Kong Chinese [9].

Cytokines are known to be important in antiviral action. Interferon (IFN)-γ from T and natural killer (NK) cells is important in driving the T helper cell type 1 (Th1) responses. It also activates monocytes and macrophages, which in turn take part in antiviral responses by producing free radicals and pro-inflammatory cytokines like tumor necrosis factor (TNF)-α. [10]. TNF-α then regulates expression of neutrophil-endothelial cell adhesion molecules and chemokines, which recruit leukocytes to the site of infection [11-13]. Thus, IFN-γand TNF-α play important role in antiviral response and inflammation.

Interleukin 10 (IL-10) is an antiinflammatory cytokine that inhibits the activation and effector function of Th1 cells, monocytes, and macrophages [14]. IL-10 appears to limit and ultimately terminate inflammatory responses by blocking the expression of a number of pro-inflammatory cytokines and chemokines [15]. In animal model, IL10 counteracts the inflammatory response by inhibiting TNF-α production and neutrophil activation, and leads to a reduction of the lung tissue injury [16]. Thus, IL-10 plays an important role in regulating many immune and inflammatory processes. Various studies showed that a high IL-10 level would result in suppression of innate host defense and lead to increasing susceptibility of the host to various microbes and death [17-19].

In this study, we hypothesized that the polymorphisms of the cytokine genes, i.e. *IFN-γ *+874A/T, *TNF-α *-308G/A, *IL-10 *-1082G/A and -592A/C, might be associated with SARS. These genes were chosen based on their functions in antiviral response and inflammation regulation that may be involved in SARS pathogenesis and their polymorphisms based on their potential regulation on gene expression (Table 1). We tested our hypotheses in 476 SARS patients and 449 healthy controls and found that polymorphism of *IFN-γ *+874A allele was associated with susceptibility to SARS in a dose-dependent manner.

**Table 1:** Polymorphisms of the genes genotyped

Genes	SNPs	rs number	References
*IFN-γ*	*IFN-γ *+874 A/T	rs2430561	[23]
*IL-10*	*IL-10 *-1082 A/G	rs1800896	[23,26-27]
	*IL-10 *-592 A/C	rs1800872	
*TNF-α*	*TNF-α *-308G/A	rs1800629	[28]

## Methods

### Patient populations

The study was approved by the Clinical Research Ethics Committee of the Institutional Review Board of the University of Hong Kong/Hospital Authority Hong Kong West Cluster and included 476 Chinese patients with SARS (201 male, mean age = 39.8 ± 15.2) and 449 ethnically matched healthy controls from Red Cross (273 male, mean age = 29.1 ± 10.4). At least 95% of the patients were documented with SARS-CoV antibody seroconversion and/or detectable SARS-CoV RNA in respiratory secretions by RT-PCR.

### Genotyping

*IFN-γ *+874A/T, *IL-10 *-1082G/A and -592A/C were genotyped by TaqMan system (Applied Biosystems, Foster City, CA, USA) as described previously [20]. *TNF-α *-308 G/A was also genotyped by TaqMan system with same condition. The sequences of the primers were 5'-CCT GGT CCC CAA AAG AAA TG-3' and 5'-TCT TCT GGG CCA CTG ACT GA-3' and the probes were 6-FAM-TTG AGG GGC ATG **G**GG ACG G-TAMRA and VIC-TTG AGG GGC ATG **A**GG ACG GG-TAMRA.

### Statistical analysis

The frequencies of genotypes and alleles of the 4 single nucleotide polymorphisms (SNPs) were compared between the SARS patients and healthy controls by 3 × 2 and 2 × 2 chi square test respectively. In case of significance, logistic regression was used for calculating OR with 95% CI and corresponding *P-*values between groups by controlling age and sex as covariables. The genotypes of all SNPs were tested for Hardy-Weinberg equilibrium (HWE) by chi square test.

## Results and discussion

Our case-control study genotyped the 4 SNPs *IFN-γ *+874A/T, *TNF-α *-308G/A, *IL-10 *-1082G/A and -592A/C in 476 Chinese patients with SARS and 449 healthy controls. The genotype distributions and allele frequencies of these 4 SNPs were shown in Table 2. The *IFN-γ *+874A allele was overrepresented in SARS patients (83.1%) when compared with the controls (66.3%) (*P *< 0.001). It was also significantly associated with susceptibility to SARS in a dose-dependent manner (*P *< 0.001), i.e. individuals with *IFN-γ *+874 AA and AT genotype had an odds ratio (OR) of 5.19 (95% CI, 2.78-9.68) and 2.57 (95% CI, 1.35-4.88) in developing SARS respectively. However, no significant correlation was observed in SNPs of *IL-10 *and *TNF-α*. All SNPs were in Hardy-Weinberg equilibrium (HWE) (*P *> 0.05) in SARS patients and controls by chi square test, except *IL-10*-592A/C.

**Table 2:** Allele frequencies and genotype frequencies in SARS patients and controls*

SNP	SARS (n = 476)	Control (n = 449)	OR (95% CI)	*P*
	Number (%)			
**Genotype**				
*IFN-γ *+874				<0.001
A/A	332 (69.8)	203 (45.2)	5.19 (2.78 – 9.68)	
A/T	127 (26.7)	189 (42.1)	2.57 (1.35 – 4.88)	
T/T	17 (3.6)	57 (12.7)	Reference	
*IL-10 *-1082				NS
A/A	439 (92.2)	411 (91.5)	..	
A/G	35 (7.4)	38 (8.5)	..	
G/G	2 (0.4)	0 (0)	..	
*IL-10 *-592				NS
A/A	244 (51.3)	209 (46.6)	..	
A/C	188 (39.5)	214 (47.7)	..	
C/C	44 (9.2)	26 (5.8)	..	
*TNF-a *-308				NS
GG	403 (84.7)	377 (83.9)	..	
GA	70 (14.7)	70 (15.6)	..	
AA	3 (0.6)	2 (0.5)	..	

**Allele**				
*IFN-γ *+874				<0.001
A	791 (83.1)	595 (66.3)	2.23 (1.75 – 2.83)	
T	161 (16.9)	303 (33.7)		
*IL-10 *-1082				NS
A	913 (95.9)	860 (95.8)	..	
G	39 (4.1)	38 (4.2)	..	
*IL-10 *-592				NS
A	676 (71.0)	632 (70.4)	..	
C	276 (29.0)	266 (29.6)	..	
*TNF-a *-308				NS
G	876 (92.0)	824 (91.8)	..	
A	76 (8.0)	74 (8.2)	..	

NS = not significant.

*P-value and OR (95% CI) were calculated with the use of logistic regression models, adjusted with sex and age.

*IFN-γ *+874A allele has been previously reported to be associated with infectious diseases such as tuberculosis, hepatitis B virus infection, and parvovirus infection [20-22], revealing its potential role of function in host defense against microbial infections. The mechanism by which the *IFN-γ *+874A/T allele influences the susceptibility to SARS may depend on its role in the regulation of IFN-γ production. The T allele of *IFN-γ *+874A/T provides a binding site for the transcription factor nuclear factor-κB (NF-κB), which is able to regulate *IFN-γ *expression [23]. It is possible that low IFN-γ production may impair their anti-viral response against SARS-CoV, rendering these individuals more susceptible to this virus infection. Our observation that *IFN-γ *+874A allele was significantly associated with SARS-CoV infection suggests a genetic risk factor for SARS. The role of IFN-γ in antiviral response against SARS-CoV has also been supported by recent studies showing that IFN-γ can inhibit the replication of SARS-CoV in combination with IFN-β *in vitro *[24,25].

*IL-10 *and *TNF-α *SNPs were also included in this study. They were chosen due to their potential regulation on protein expression level [26-28]. However, our present data did not show any significant association of these SNPs with SARS (Table 2). Nevertheless, we cannot exclude the role of *IL-10 *and *TNF-α *as the susceptibility genes for SARS, because other SNPs in these 2 genes may also be involved in gene expression regulation. Further association studies on other SNPs, which could alter the gene expression level are required to ascertain the relationship of *IL-10 *and *TNF-α *in SARS.

We have also compared the genotype and allele frequencies of all the polymorphisms between the death group and survival group of the SARS patients (Table 3). However, no significant association was established.

**Table 3:** Genotype frequencies among survival and death SARS cases

SNP	Death (n = 57)	Survival (n = 415)	*P*
	Number (%)		
**Genotype**			
*IFN-γ *+874			NS
A/A	41 (71.9)	289 (69.6)	
A/T	13 (22.8)	112 (27.0)	
T/T	3 (5.3)	14 (3.4)	
*IL-10*-1082			NS
A/A	52 (91.2)	383 (92.3)	
A/G	4 (7.0)	31 (7.5)	
G/G	1 (1.8)	1 (0.2)	
*IL-10*-592			NS
A/A	28 (49.1)	214 (51.6)	
A/C	21 (36.8)	165 (39.8)	
C/C	8 (14.0)	36 (8.7)	
*TNF-a*-308			NS
GG	46 (80.7)	353 (85.1)	
GA	11 (19.3)	59 (14.2)	
AA	0 (0)	3 (0.7)	

NS = not significant.

## Conclusion

We demonstrated that *IFN-γ *+874A allele was significantly associated with SARS susceptibility in a dose dependent manner. Due to its role in regulating IFN-γ expression [15], this allele may be involved in the pathogenesis of SARS by altering the IFN-γ production.

## Competing interests

The author(s) declare that they have no competing interests.

## Authors' contributions

WPC and WKEI: Genotyping, data analyses, drafting the manuscript

GHWT: Genotyping

MWN and WHSW: Data analyses, drafting the manuscript

HKWL, RWHY, EYC, KLA, EYTC: Sample collection, revising for medical content

WL and JSMP: Sample collection, providing virological data

YLL: Study design, conception and co-ordination, drafting the manuscript

All authors contributed to writing of the final manuscript

All authors read and approved the final manuscript

## Pre-publication history

The pre-publication history for this paper can be accessed here:


